# Dendritic cell-based immunotherapy modulates the systemic inflammatory profile in a 4T1 breast cancer model

**DOI:** 10.37349/etat.2026.1002393

**Published:** 2026-08-05

**Authors:** Tauana Christina Dias, Jéssica Ferreira Vieira, Katiane Tostes, Polyana Barbosa Silva, Angela Maria Moed Lopes, Gabriela Karam Rebolho, Eddie Fernando Candido Murta, Lidia Maria Rebolho Batista Arantes, Márcia Antoniazi Michelin

**Affiliations:** Fondazione Policlinico Universitario Agostino Gemelli IRCCS, Italy; ^1^Oncology Research Institute (Instituto de Pesquisa em Oncologia, IPON), Federal University of the Triângulo Mineiro (Universidade Federal do Triângulo Mineiro, UFTM), Uberaba 38025-350, Minas Gerais, Brazil; ^2^Molecular Oncology Research Center, Barretos Cancer Hospital, Barretos 14784-400, São Paulo, Brazil; ^3^Department of Gynecology and Obstetrics, UFTM, Uberaba 38025-440, Minas Gerais, Brazil; ^4^Immunology Discipline, UFTM, Uberaba 38025-050, Minas Gerais, Brazil

**Keywords:** dendritic cell, immunotherapy, breast cancer, 4T1, mice, inflammation

## Abstract

**Aim:**

Breast cancer remains a major cause of cancer-related mortality in women, particularly in advanced stages where therapeutic options are limited. While immune checkpoint inhibitors (ICIs) have improved outcomes in a subset of patients, many do not respond, highlighting the need for alternative immunotherapeutic strategies. This study evaluated the effect of dendritic cell (DC)-based immunotherapy on tumor growth and on the inflammatory profile of peritoneal myeloid cells in a 4T1 murine breast cancer model.

**Methods:**

BALB/c mice bearing 4T1 breast tumors were treated with bone marrow-derived DC-based immunotherapy. Tumor volume was monitored over time, and CD14^+^ cells obtained from peritoneal lavage were analyzed by flow cytometry for the cytokines IL-12, IL-17, and TNF-α and the transcription factors RORγT and GATA3.

**Results:**

DC-based immunotherapy was associated with a non-significant trend toward reduced tumor volume and a marked suppression of key proinflammatory cytokines: IL-12 (*P* < 0.0005), IL-17 (*P* < 0.0001), and TNF-α (*P* < 0.0001). Expression of the transcription factors RORγT and GATA3, associated with Th17 and Th2 differentiation, was also downregulated (*P* < 0.0001). These immunological effects were observed in CD14^+^ myeloid cells from the peritoneal compartment.

**Conclusions:**

DC-based immunotherapy modulates the systemic/peritoneal inflammatory profile and attenuates tumor-promoting inflammation. This strategy may offer therapeutic benefit for patients with breast cancer who are unresponsive to conventional or ICI-based treatments and supports its further evaluation in translational studies.

## Introduction

According to GLOBOCAN estimates for breast cancer, over 2,296,840 new cases were diagnosed in 2022, making it the most prevalent and incident type of tumor among women worldwide [1]. Despite being the most common cancer among women worldwide, the treatment landscape has undergone significant changes in recent years. These changes can be attributed to various factors, including the emergence of molecular markers, advancements in surgical treatments, radiotherapy, chemotherapy, and hormone therapy. Moreover, the introduction of immunotherapy has provided a new perspective for women affected by this tumor type [2].

The use of immunotherapy in breast cancer treatment has been evolving, with research into its various applications steadily expanding. One significant advancement is the development of immune checkpoint inhibitors (ICIs), a form of immunotherapy that targets the programmed death-1 (PD-1) and programmed death-ligand 1 (PD-L1) pathways. This breakthrough has transformed the treatment of solid tumors and demonstrated promising results in improving patient outcomes [3].

In the field of immunotherapy, dendritic cell (DC) therapy provides an alternative treatment option for patients who have not responded to conventional therapies. This approach leverages the unique ability of DCs to identify and eliminate neoplastic cells through immunological surveillance, which involves the coordination of innate and adaptive immunity, utilizing components such as CD8^+^ T lymphocytes, CD4^+^ Th1 lymphocytes, natural killer (NK) cells, DCs, and phagocytic cells (monocytes and macrophages). Together, these immune cells establish a targeted response specifically directed against neoplastic cells [3–7].

DCs, classified as professional antigen-presenting cells (APCs), can capture tumor antigens, process and present them through the major histocompatibility complex (MHC) classes I and II to CD8^+^ and CD4^+^ T lymphocytes, respectively. The presentation of tumor antigens to CD4^+^ T lymphocytes leads to their differentiation into Th1 lymphocytes, which secrete cytokines that influence other components of the immune system, including macrophages [8–11].

Macrophages, like Th1 and Th2 lymphocytes, exhibit two activation profiles: M1 and M2. M1 macrophages are polarized via the Th1 pathway through IFN-γ, leading to the secretion of pro-inflammatory cytokines (e.g., TNF-α, IFN-γ, IL-1, IL-6, IL-12), reactive oxygen species (ROS), and nitric oxide (NO), which are critical for inflammation and antitumor activity [10, 12]. Conversely, Th2 lymphocytes secrete IL-4 and IL-13, polarizing M2 macrophages to produce IL-10, IL-4, arginase, prostaglandin, TGF-β, and VEGF, mediators that support tumor progression [10].

In addition to cytokines, transcription factors significantly impact the tumor process. Although their expression was initially thought to be restricted to hematopoietic lymphoid lineages, the presence of FOXP3, GATA3, and T-bet has been observed in other cells, contributing to immunosuppression and tumor growth [6, 13], as has the modulation of immune-cell infiltration within the tumor microenvironment [14]. RORγT is the lineage-defining transcription factor of Th17 cells, driving IL-17 production [15], which in the breast tumor microenvironment acts predominantly as a pro-tumoral mediator, promoting tumor cell invasiveness [16]. Although most studies of DC-based immunotherapy in breast cancer have focused on the local tumor microenvironment, tumor progression in aggressive models such as 4T1 is shaped by the host’s broader inflammatory state, which extends beyond the tumor site. Myeloid cells, including monocytes and macrophages, are central to this systemic inflammatory compartment and can either support or restrain tumor growth depending on their activation profile. Characterizing how DC immunotherapy modulates these cells outside the tumor itself may therefore reveal effects that are not captured by intratumoral analysis alone. In this context, the present study evaluated the impact of DC-based immunotherapy on the inflammatory profile of CD14^+^ myeloid cells obtained from the peritoneal compartment of mice bearing 4T1-induced breast tumors, with the aim of clarifying how this therapeutic approach reshapes the systemic immune response rather than the tumor microenvironment alone.

## Materials and methods

**Figure 1:**
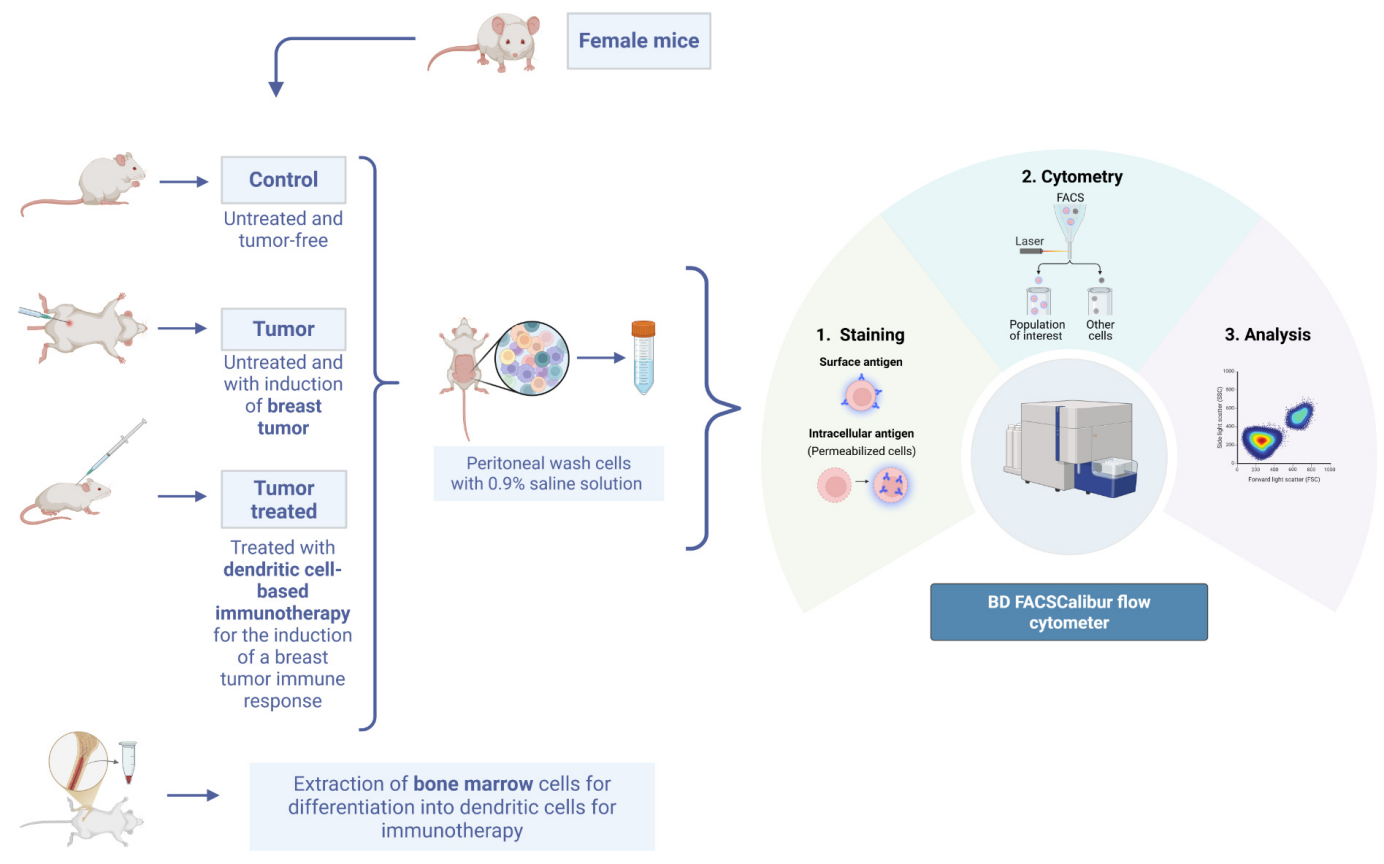
**Experimental design. BALB/c mice were divided into three groups: control (tumor-free), tumor (4T1 cell-induced), and tumor + DC (treated with dendritic cell-based immunotherapy).** Bone marrow-derived DCs were generated in vitro and administered to the treatment group. Peritoneal lavage was performed and cells were analyzed by flow cytometry. Created in BioRender. Arantes, L. (2026) https://BioRender.com/hydw939.

A total of 30 randomly selected 8-week-old female BALB/c wild-type mice were used in a 28-day experimental protocol (Figure 1). Animals were housed in plastic cages under a 12-hour light/dark cycle at 21 ± 3°C, with food and water provided ad libitum. Mice were randomly assigned to three experimental groups: the control group (healthy mice treated with 0.9% saline solution), the tumor group (mice inoculated with 4T1 breast tumor cells), and the tumor + DC group (mice inoculated with 4T1 cells and treated with DC-based immunotherapy). Each group consisted of 10 animals divided equally across two independent experimental replicates (*n* = 5 per group per replicate). An additional 10 mice were used exclusively for bone marrow extraction to generate DCs. Animals from different experimental groups were housed in separate cages to avoid cross-exposure. All animals were obtained from the Central Animal Facility of the Federal University of Triângulo Mineiro, were not subjected to genotyping, and were of the same genetic background and age-matched.

At the end of the experimental period, animals were euthanized by an overdose of ketamine (50 mg/kg) and xylazine (15 mg/kg). Peritoneal lavage was then performed on all groups by injecting 5 mL of 0.9% saline solution into the peritoneal cavity to recover resident monocytes/macrophages.

### Tumor induction of breast cancer model

The 4T1 cells were cultured in Roswell Park Memorial Institute medium (RPMI-1640; Sigma-Aldrich^®^, St. Louis, MO, USA) and incubated at 37°C in a humidified atmosphere with 5% CO_2_. The medium was supplemented with 0.24% HEPES, sodium bicarbonate (NaHCO_3_), 10% fetal bovine serum (FBS), 1% streptomycin, 1% L-glutamine (200 mM), 0.1% sodium pyruvate, and 0.1% β-mercaptoethanol. After the culture period, cells were washed with 0.9% saline solution and centrifuged at 290× *g* for 10 minutes at 4°C before subcutaneous inoculation into female BALB/c mice. Tumor size was assessed by measuring the mammary tumor in the cranio-caudal and latero-lateral directions with a 150 mm universal analog caliper, beginning when the tumor became palpable and every 2–3 days thereafter until the day before euthanasia. Tumor volume was calculated according to Faustino-Rocha et al. [17, 18] using the formula V = (W^2^ × L)/2, where V is the tumor volume (mm^3^), W is the tumor width (smallest diameter), and L is the tumor length (largest diameter).

### DC immunotherapy

DC immunotherapy consisted of three vaccine doses. Each dose contained 5 × 10^6^ bone marrow-derived DCs, obtained from the femur and tibia of three female BALB/c mice, suspended in 50 μL of 0.9% saline solution, and was administered subcutaneously in the dorsal region once a week for three consecutive weeks. The DC generation protocol used in this study is based on the autologous DC method patented by our group [19], and the 5 × 10^6^ DC dose was selected based on our group’s previous experimental work in this model [7]. The cells were plated and cultured in Iscove’s Modified Dulbecco’s Medium (IMDM; Sigma-Aldrich^®^, St. Louis, MO, USA) supplemented with 0.1 mM vitamins, 2 mM L-glutamine, 100 μg/mL gentamicin, 1 mM sodium pyruvate, and 5% FBS, and incubated at 37°C in a humidified atmosphere with 5% CO_2_. For differentiation of the bone marrow-derived precursor cells, 10 ng/μL of granulocyte-macrophage colony-stimulating factor (GM-CSF) and 10 ng/μL of IL-4 were added on day 1. On day 5, cells were stimulated with tumor lysate from 4T1 cells and TNF-α. GM-CSF, IL-4, and TNF-α were obtained from BD Pharmingen™ (BD Biosciences, San Diego, CA, USA). After 48 h of incubation, the differentiated DCs were washed and resuspended in 0.9% saline solution. In the tumor-treated (tumor + DC) group, the first dose was administered one week after 4T1 tumor induction, characterizing a therapeutic vaccination protocol.

### Characterization of immune cells by flow cytometry

Macrophages/monocytes were obtained through peritoneal lavage by injecting 5 mL of 0.9% saline solution into the abdomen of the mice. For the analysis, the cells were washed twice with RPMI-1640 medium and centrifuged at 657× *g* for 10 minutes at 4°C. Cells were counted using a Neubauer chamber and labeled with the extracellular marker anti-CD14 (FITC, clone M5E2, BD Biosciences) to identify the murine monocyte/macrophage population. After 30 minutes of incubation, the cells were washed and prepared for intracellular staining with IL-12 (PE, clone C8.6), IL-17 (PerCP-Cy5.5, clone eBio64DEC17), TNF-α (PE, clone MAb11), and the transcription factors RORγt (PE, clone Q21-559) and GATA3 (PE, clone L50-823) (all BD Biosciences). To minimize nonspecific binding, cells were pre-incubated with anti-mouse IgG2a (clone eB149/10H5) and IgG2b (clone eB149/10H9) antibodies. Corresponding isotype controls—rat IgG2a PE (eBR2a) and rat IgG2b FITC (eB149/10H9)—were used to assess background staining for PE- and FITC-conjugated antibodies (Figure S4). For the IL-17 marker (PerCP-Cy5.5), no isotype control was available; therefore, analysis was performed exclusively based on the median fluorescence intensity (MFI) of the CD14^+^ population, allowing comparison between groups without inference regarding the percentage of positive cells. Samples were analyzed using a BD FACSCalibur flow cytometer (BD Biosciences), and data were processed using FlowJo software. The gating strategy began with selection of viable cells based on FSC-A and SSC-A to exclude debris, followed by singlet selection (FSC-A vs FSC-H). CD14^+^ monocytes were then identified (CD14-FITC vs SSC-H), and this gated population was used for downstream cytokine analysis (Figure S1). The same gating and compensation strategy was applied identically across all samples and the two independent biological replicates.

### Statistical analysis

Data were analyzed using one-way ANOVA followed by Tukey’s post hoc test for multiple group comparisons, as the data were normally distributed and met assumptions of homogeneity of variances. Tumor volume data were tested for normality using the Shapiro-Wilk test. Longitudinal tumor volume was analyzed by two-way ANOVA considering treatment and time as factors, accounting for repeated measurements over time, followed by Bonferoni multiple-comparison test, replacing the paired *t*-tests used in the original submission. Cytokine and transcription-factor results are expressed as mean ± standard deviation (SD); tumor volume is shown as mean ± standard error of the mean (SEM). Statistical significance was defined as *P* < 0.05. All analyses were performed using GraphPad Prism version 8.0 (GraphPad Software, Inc., La Jolla, CA, USA).

## Results

### DC immunotherapy and longitudinal tumor volume assessment

**Figure 2:**
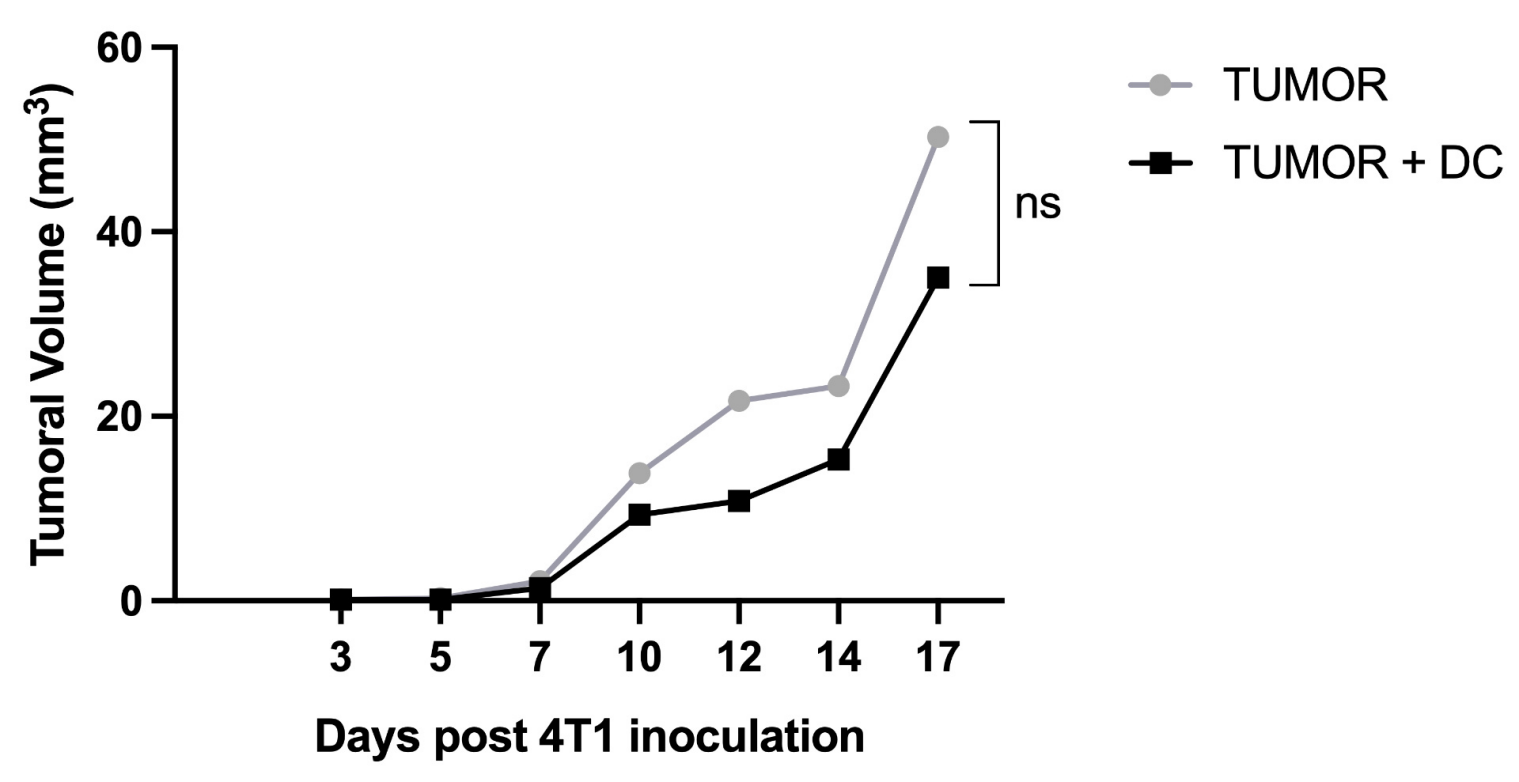
**Tumor volume progression in 4T1 breast cancer-bearing mice.** Mice treated with dendritic cell immunotherapy (tumor + DC) showed a non-significant trend toward lower tumor volume compared to the untreated tumor group (two-way ANOVA, effect of treatment *P* = 0.392 (*n* = 5 per group). DC: dendritic cell.

Tumor growth monitoring revealed that mice treated with DC immunotherapy (tumor + DC) showed a trend toward lower tumor volume compared to untreated tumor-bearing mice (tumor group); however, this difference was not statistically significant. Two-way ANOVA showed a significant effect of time (*F*(6,53) = 3.00; *P* = 0.013), reflecting tumor growth, but no significant effect of treatment (*F*(1,53) = 0.74; *P* = 0.392) and no significant treatment × time interaction (*F*(6,53) = 0.12; *P* = 0.994); per-timepoint comparisons were also non-significant (all *P* > 0.27) (Figure 2). One animal in the tumor group died after day 10 and was analyzed using its available time points.

### Immunotherapy with DCs altered the cytokine profile

**Figure 3:**
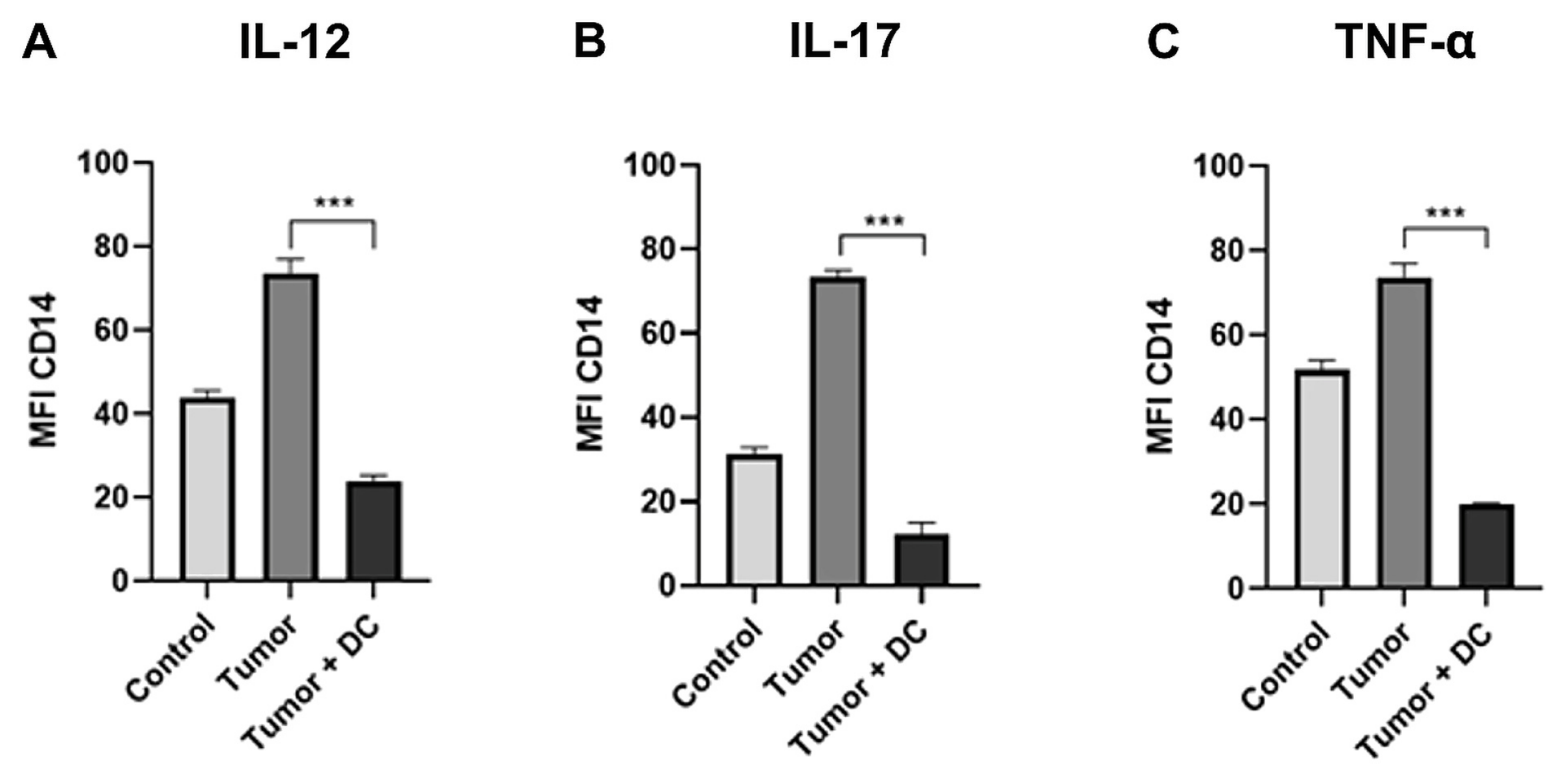
**Expression of pro-inflammatory cytokines.** Median fluorescence intensity (MFI) of IL-12 (**A**), IL-17 (**B**), and TNF-α (**C**) in CD14^+^ monocytes/macrophages from peritoneal lavage. Tumor-bearing mice treated with DCs showed significantly decreased cytokine expression compared to the untreated tumor group (***: *P* < 0.001). DC: dendritic cell.

Immunotherapy with DCs altered the cytokine profile of CD14^+^ monocytes/macrophages obtained from the peritoneal wash after euthanasia, analyzed by flow cytometry immediately after collection. Cytokine expression was evaluated using the MFI. A significant reduction was observed in IL-12 (*P* < 0.0005), IL-17 (*P* < 0.0001), and TNF-α (*P* < 0.0001) in the tumor-treated group compared to the untreated group (Figure 3; Figure S2).

### Immunotherapy with DCs reduced GATA3 and RORγT expression

**Figure 4:**
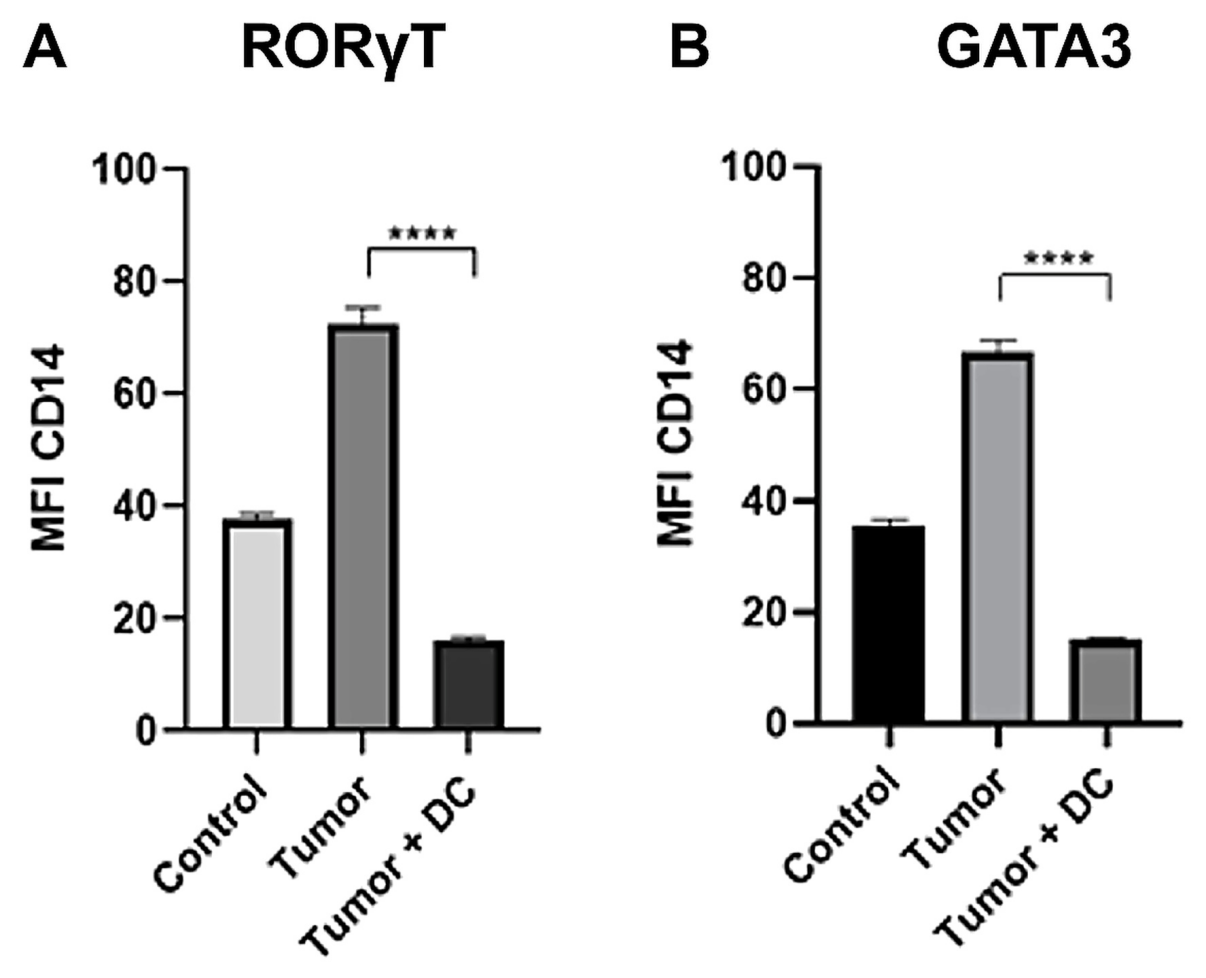
**Expression of transcription factors.** MFI of RORγT (**A**) and GATA3 (**B**) in CD14^+^ cells. Treatment with DC immunotherapy resulted in significantly lower expression of these transcription factors compared to the untreated tumor group (****: *P* < 0.0001). DC: dendritic cell; MFI: median fluorescence intensity.

In the same samples, the expression of the transcription factors GATA3 and RORγT was evaluated based on MFI values. A significant reduction in their expression was observed in the group treated with DC immunotherapy compared to the untreated tumor group (*P* < 0.0001) (Figure 4; Figure S3).

## Discussion

After several years of decline, breast cancer mortality has shown an upward trend, currently occupying the second position among the leading causes of cancer-related deaths. This increase may be associated with the COVID-19 pandemic, which reduced screening exams crucial for early detection [20, 21]. Consequently, diagnoses were often made at more advanced stages, resulting in poorer clinical outcomes [21]. Given this scenario, studies using experimental models play a fundamental role in understanding advanced-stage breast cancer behavior and in developing new therapeutic approaches for patients who do not respond to conventional therapies [22]. ICIs have emerged as an alternative to conventional modalities such as chemotherapy, radiotherapy, and surgery, employing strategies focused on immune system modulation [23, 24].

However, despite advances in immunotherapy, some patients exhibit little to no response to ICIs. These non-responders present a significant challenge [25], making it essential to pursue alternative immunotherapies capable of modulating the immune system [26].

In this context, cellular therapies have gained prominence as a promising option for patients who do not respond to ICIs [27]. Among these, DC immunotherapy stands out for its role in modulating the immune system, and it can synergize with ICI treatment [28].

DC vaccines function by priming the immune system to recognize and attack tumor cells. The cells are previously induced to differentiate into DCs with an anti-tumor profile, and upon administration they stimulate the immune system to fight the tumor [29].

In this preclinical study conducted on BALB/c mice, we observe a significant reduction in IL-12, IL-17, and TNF-α levels in the tumor-treated group compared to the untreated group. The overall reduction of these cytokines is important considering the concept of the tumor escape mechanism associated with chronic inflammation [30]. High levels of IL-17A are associated with more aggressive and invasive breast cancer; the reduction of IL-17 may contribute to antitumor effects, as a decrease in this cytokine reduces associated processes such as angiogenesis, tumorigenesis, inhibition of apoptosis, and migration [16, 30, 31]. Although there was no statistically significant difference in tumor growth between the tumor-only group and the tumor + DC group, the immunological findings were robust. The immunological findings were substantial, providing insight into the systemic behavior of CD14^+^ cells from peritoneal lavage following DC immunotherapy. These findings corroborate those from other cell types and tissues that point to improved immune system performance when DC immunotherapy is used to prevent or treat 4T1-induced tumors [7, 8, 32].

Similarly to IL-17, TNF-α expression was reduced by DC-based immunotherapy. This reduction also contributes to decreased proliferation, angiogenesis, and migration, as these processes are partly regulated by TNF-α [16, 30, 31, 33].

The regulation of the immune system by DCs can also lead to reduced expression of IL-12, a cytokine classically associated with antitumor activity [34]. However, reduced expression does not necessarily indicate a compromised immune response [35, 36]; it may reflect attenuation of the local inflammatory milieu while systemic levels remain elevated [37]. In the present study, the peritoneal lavage of BALB/c mice was examined. We note that peritoneal cells represent a systemic/peritoneal compartment rather than the tumor microenvironment itself, and the cytokine findings should be interpreted accordingly. The reduction in IL-12, along with the decrease in other cytokines, highlights the importance of controlling chronic inflammation [38, 39], which in turn reduces tumor-induced immunosuppressive mechanisms and reflects the balance necessary for antitumor activity [39].

The critical balance between acute and chronic inflammation can also be illustrated by the transcription factors RORγT and GATA3, which showed a significant reduction in treated mice. RORγT is the lineage-defining transcription factor of Th17 cells, driving IL-17 production [15]. Although not classically associated with the myeloid compartment, it was deliberately assessed here, and its significant reduction in the CD14^+^ compartment following DC-based immunotherapy merits further investigation.

Similarly, GATA3 is a lineage-defining transcription factor of the T-cell/Th2 compartment [40] and is therefore not typically regarded as a monocyte factor; nonetheless, its expression was significantly reduced in the CD14^+^ population of the DC-treated group. This reduction in GATA3 expression may be associated with decreased macrophage polarization toward the M2 profile and lower production of cytokines involved in tumor progression, such as IL-4 and IL-13 [41]; however, this link remains associative, as macrophage polarization was not directly assessed here.

In addition to the flow-cytometry approach employed in this study, the incorporation of complementary methodologies in future research would facilitate a more comprehensive characterization of the systemic effects of DC-based immunotherapy. Specifically, the implementation of functional cellular assays and proteomic profiling of CD14^+^ myeloid cells holds potential in elucidating the cellular response to treatment at the functional and protein-expression levels, extending beyond the cytokine and transcription-factor signals that have been assessed in this study. Furthermore, the potential of spectral and hyperspectral imaging techniques, which have demonstrated efficacy in computer-aided detection of breast cancer [42], to provide complementary, label-free assessments of the host response warrants further investigation. A collaborative effort between these approaches would facilitate the delineation of the systemic immunomodulatory footprint of DC immunotherapy and the role of peripheral CD14^+^ cells in this response.

### Limitations

Several limitations should be acknowledged. First, the immunological analyses were performed on CD14^+^ cells recovered from peritoneal lavage, which represents a systemic/peritoneal compartment rather than the tumor microenvironment; tumor-infiltrating and tumor-draining lymph node cells were not analyzed. Second, the difference in tumor volume between groups did not reach statistical significance with the available sample size and should be regarded as a trend. Third, T-cell subsets (CD4, CD8, and Treg) were not characterized, and macrophage polarization was not confirmed with M1/M2 markers (e.g., CD86, CD206). Fourth, DC maturation markers (CD80, CD86, MHC-II) were not assessed. Finally, IL-17 was analyzed using MFI without an isotype control, so its results reflect relative between-group differences only and blinding of the outcome assessors during tumor measurement and flow cytometry analysis was not formally documented. These points define priorities for future studies, including intratumoral immune profiling, histopathological assessment of proliferation and angiogenesis, apoptosis assays, and validation of inflammatory signaling pathways such as NF-κB.

### Conclusion

In conclusion, this study highlights the therapeutic potential of DC-based immunotherapy as an alternative strategy for breast cancer, particularly where conventional treatments or ICIs are ineffective. The observed trend toward reduced tumor volume, which did not reach statistical significance, together with the significant decrease in pro-inflammatory cytokines such as IL-12, IL-17, and TNF-α, suggests a capacity of DCs to modulate the inflammatory response and may help disrupt tumor-promoting inflammatory pathways. Moreover, the downregulation of RORγT and GATA3 suggests that DC immunotherapy may influence key regulators of immune polarization and chronic inflammation, even in the continued presence of CD14^+^ myeloid cells. These findings suggest that immune modulation by DCs can alter the systemic/peritoneal inflammatory profile and may help attenuate immunosuppressive signaling and rebalance pro- and anti-inflammatory responses; intratumoral confirmation remains to be established. Collectively, these results provide a preclinical rationale for further investigation of DC-based vaccines in breast cancer, particularly in patients with advanced disease or resistance to standard therapies.

## Abbreviations

DC: dendritic cell
FBS: fetal bovine serum
ICI: immune checkpoint inhibitor
MFI: median fluorescence intensity
MHC: major histocompatibility complex

## Supplementary materials

The supplementary figures for this article are available at: https://www.explorationpub.com/uploads/Article/file/1002393_sup_1.pdf.

## Declarations

### Availability of data and materials

The datasets analyzed for this study are available from the corresponding author upon reasonable request.

### Copyright

© The Author(s) 2026.
