# Supplementary material for: Dendritic cell-based immunotherapy modulates the systemic inflammatory profile in a 4T1 breast cancer model
Source: Explor Target Antitumor Ther. 2026 Aug 5;7:1002393. doi: 10.37349/etat.2026.1002393 (PMC13439114; doi:10.37349/etat.2026.1002393)
Supplement: Supplementary file 1 [file 1002393_sup_1.pdf]

Supplementary Materials

Dendritic Cell-Based Immunotherapy Modulates the Systemic Inflammatory Profile in a 4T1 Breast Cancer Model

Manuscript ID: ETAT-2026-00066-R1

Supplementar Figure 1

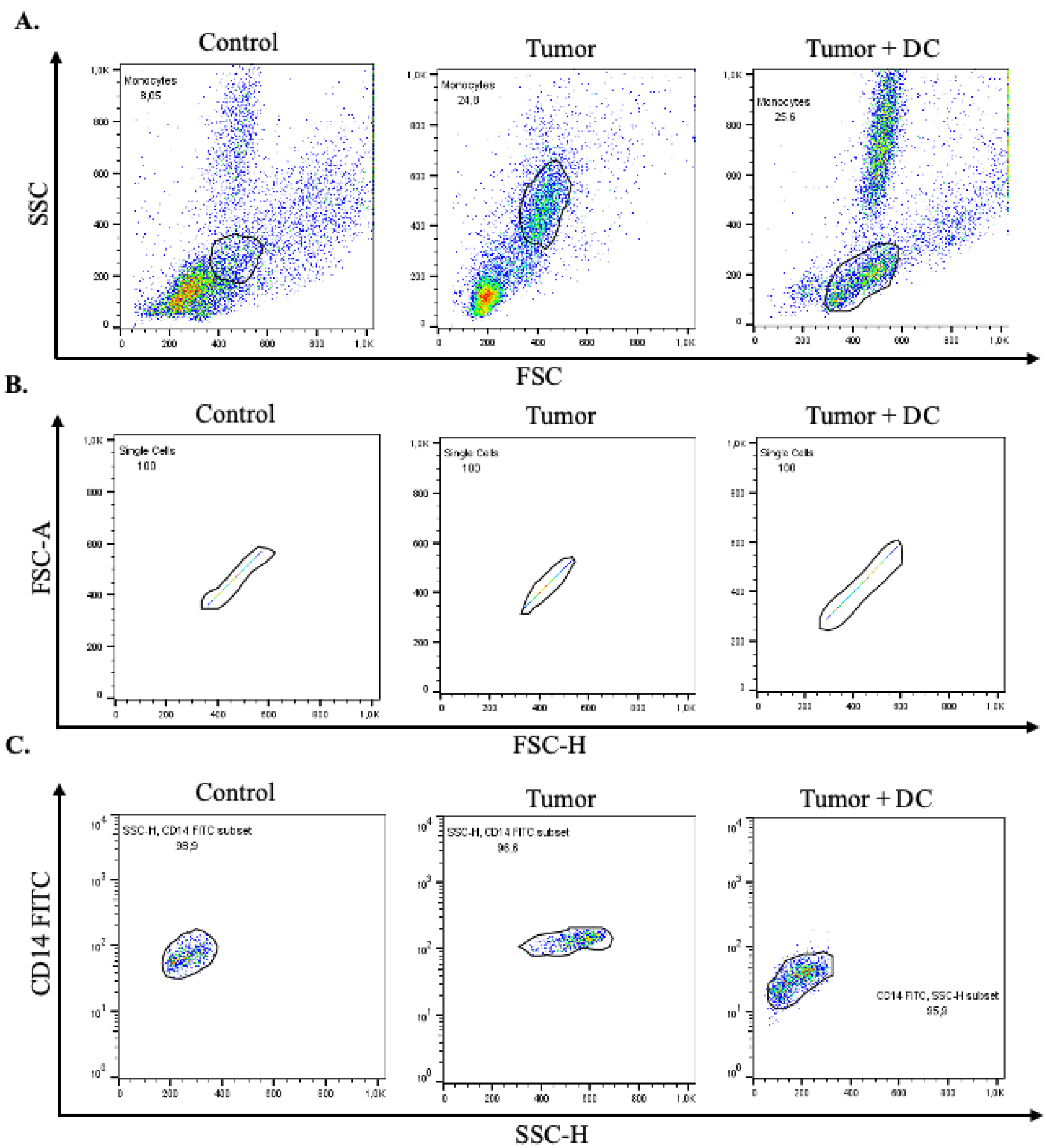

Supplementary Figure 1. Gating strategy used for the flow cytometry analysis of peritoneal lavage cells from the Control, Tumor, and Tumor + DC groups. (**A**) An initial gate was set on the region corresponding to monocytes on the basis of cell size and internal complexity, using the FSC and SSC parameters. (**B**) Doublets were subsequently excluded on an FSC-H versus FSC-A plot. (**C**) Within the singlet population, $CD14^+$ cells were selected, and this gate was used for all subsequent MFI determinations. Plots are representative of two independent experiments (*n* = 2 biological replicates per group), and the same gating strategy was applied to all groups. DC: dendritic cell; FSC: forward scatter; FSC-A: forward scatter area; FSC-H: forward scatter height; MFI: median fluorescence intensity; SSC: side scatter.

Supplementary Figure 2

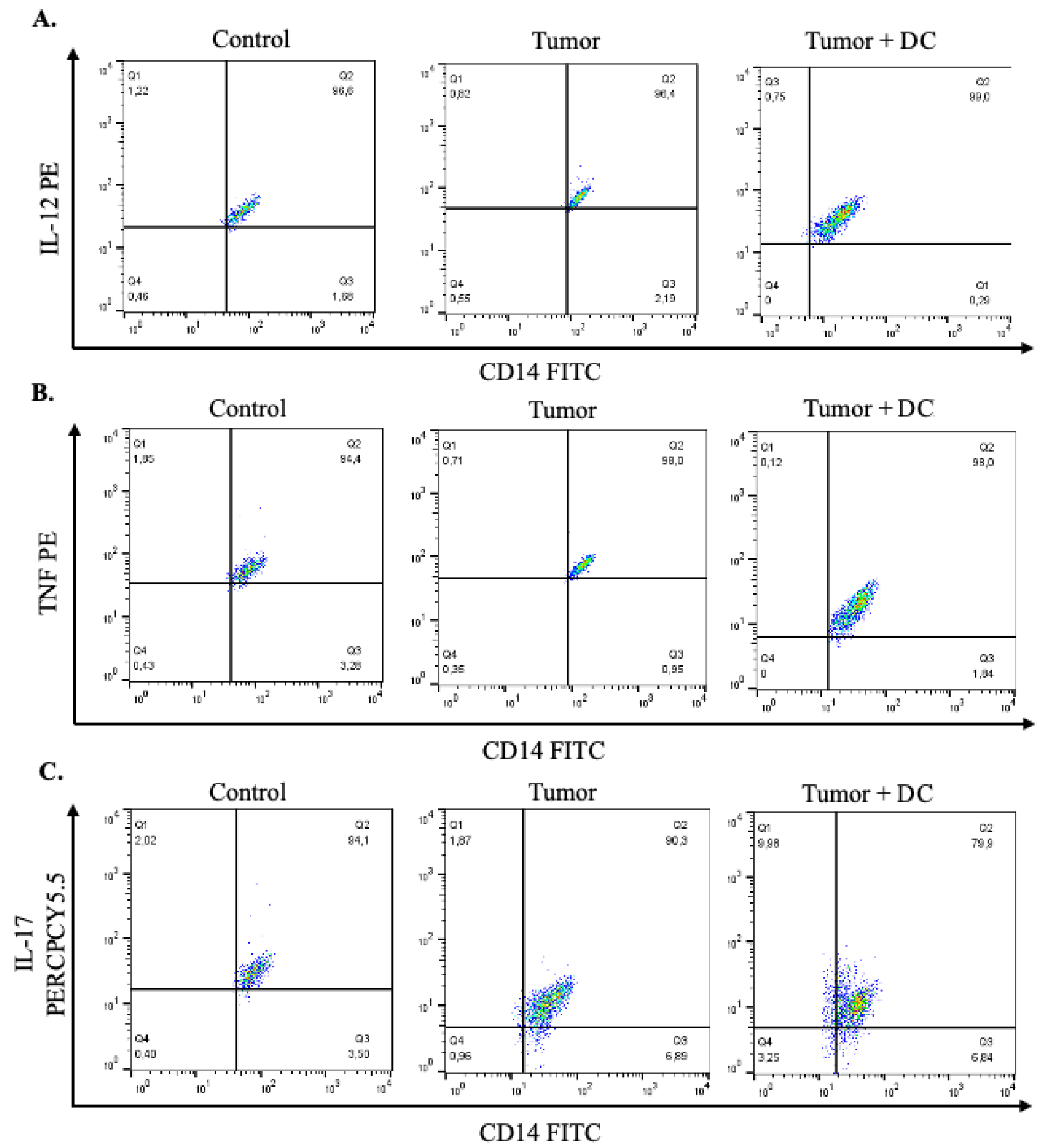

Supplementary Figure 2. Representative dot plots of cytokine expression in CD14$^+$ peritoneal monocytes from the Control, Tumor, and Tumor + DC groups. (**A**) IL-12 (PE), (**B**) TNF (PE), and (**C**) IL-17 (PerCP-Cy5.5) were assessed within the CD14$^+$ population defined by the gating strategy shown in Supplementary Figure 1. For each cytokine, the double-positive population (Q2, CD14$^+$cytokine$^+$) was delimited and its MFI was used to compare the relative expression between groups. Identical gating and compensation settings were applied to all samples. One representative plot per group is shown; plots are representative of two independent experiments (n = 2). DC: dendritic cell; IL: interleukin; MFI: median fluorescence intensity; PE: phycoerythrin; PerCP-Cy5.5: peridinin-chlorophyll protein-cyanine 5.5; TNF: tumor necrosis factor.

Supplementary Figure 3

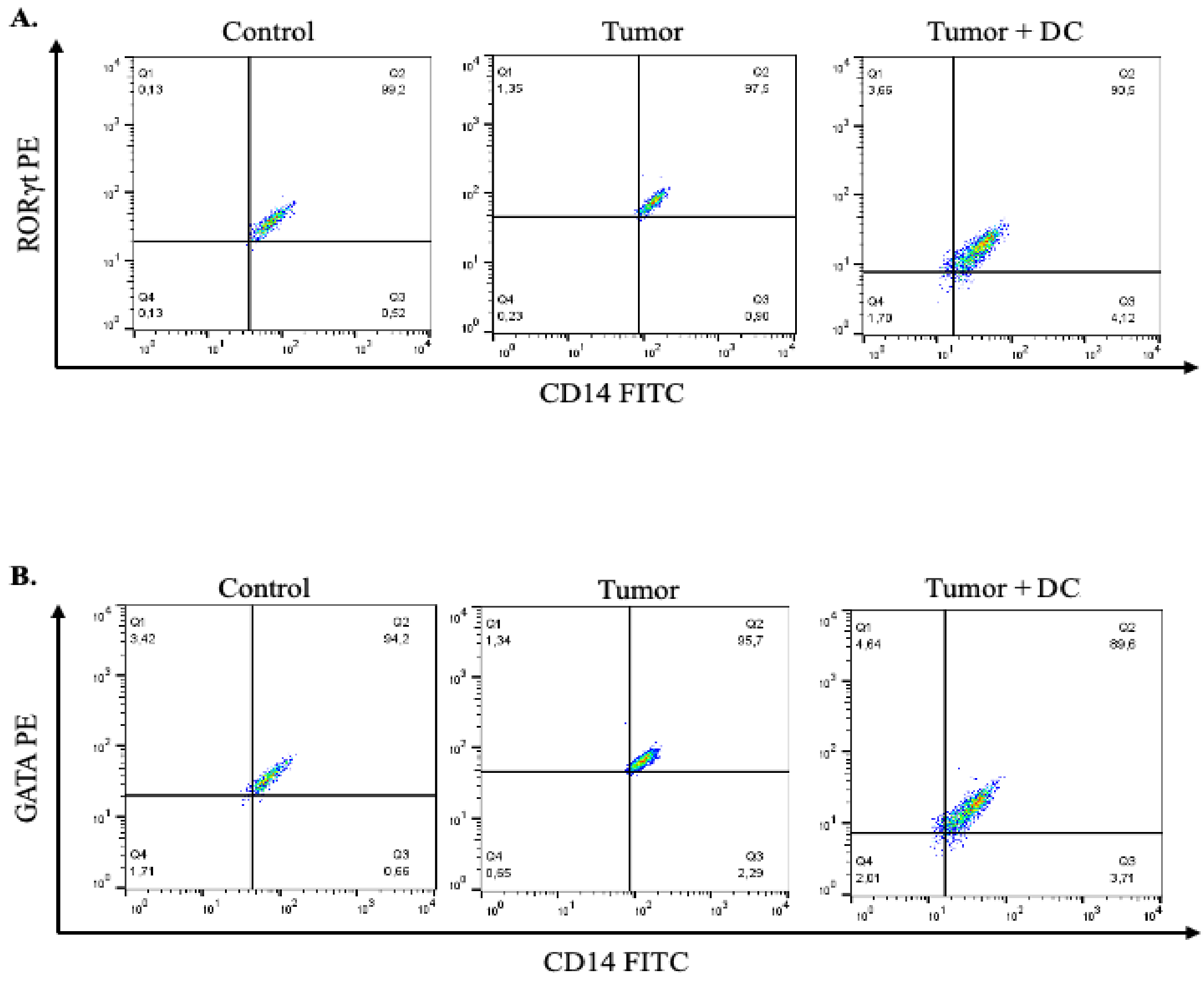

Supplementary Figure 3. Representative dot plots of transcription factor expression in CD14^{+} peritoneal monocytes from the control, tumor, and tumor + DC groups. (**A**) RORγt (PE) and (**B**) GATA3 (PE) were assessed within the CD14^{+} population defined by the gating strategy shown in Supplementary Figure 1. For each transcription factor, the double-positive population (Q2, CD14^{+}TF^{+}) was delimited and its MFI was determined. Identical gating and compensation settings were applied to all samples. One representative plot per group is shown; plots are representative of two independent experiments (n = 2). DC: dendritic cell; GATA3: GATA-binding protein 3; MFI: median fluorescence intensity; PE: phycoerythrin; RORγt: retinoic acid receptor-related orphan receptor gamma t; TF: transcription factor.

Supplementary Figure 4

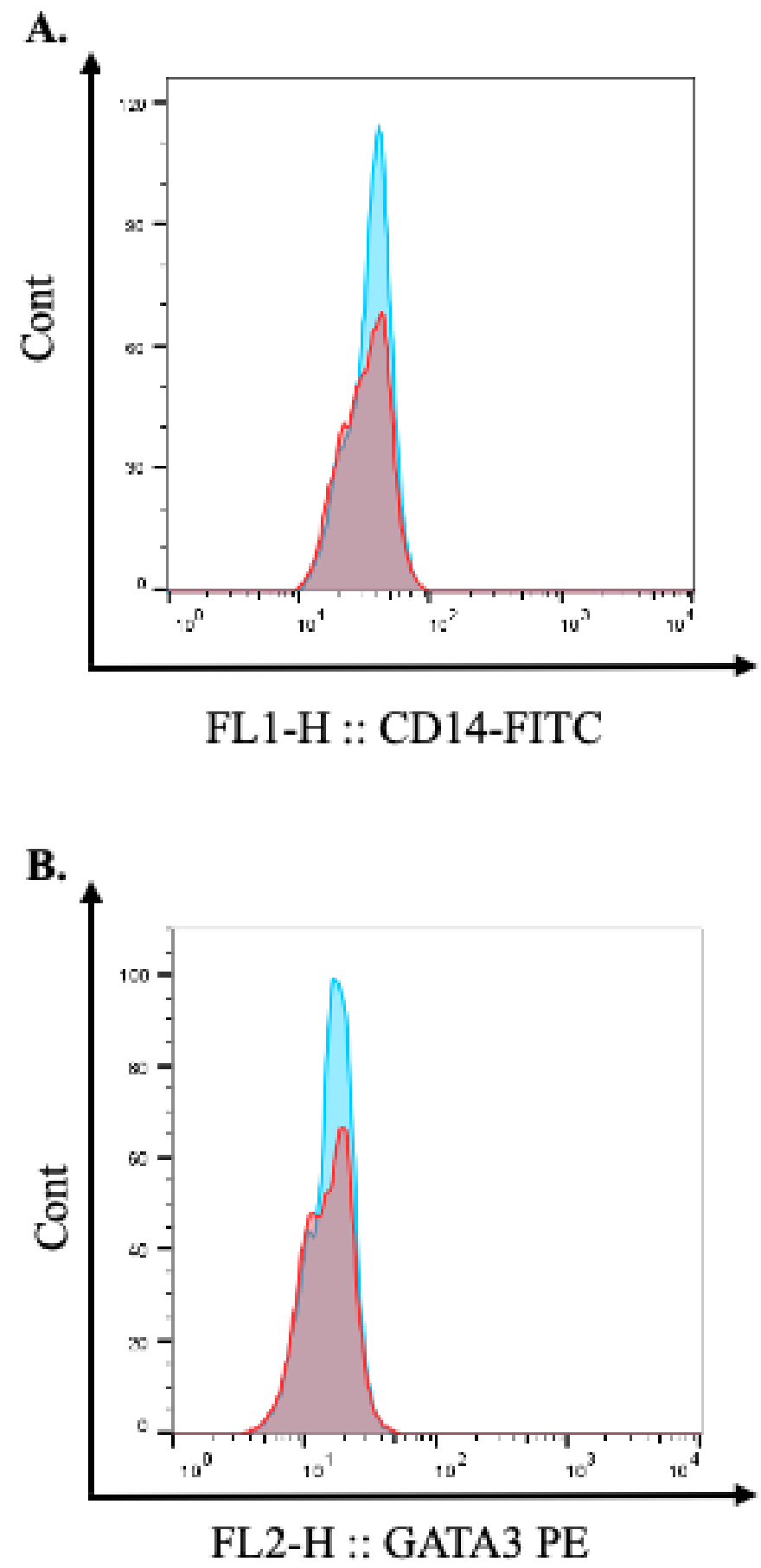

Supplementary Figure 4. Histogram overlays of the fluorescence intensity obtained with the specific antibodies (red) and with the corresponding isotype controls (blue), within the $CD14^{+}$ gated population. (**A**) CD14-FITC and the rat IgG2b-FITC isotype control (FL1-H channel). (**B**) GATA3-PE and the rat IgG2a-PE isotype control (FL2-H channel). In both panels, the isotype controls showed low background fluorescence, supporting the specificity of the staining obtained with the corresponding antibodies. Histograms are from one representative experiment. FITC: fluorescein isothiocyanate; GATA3: GATA-binding protein 3; Ig: immunoglobulin; PE: phycoerythrin.
